# Dental Education of the Public

**Published:** 1894-03

**Authors:** 


### Editorial, Etc.

DENTAL EDUCATION OF THE PUBLIC.—Tne timeliness of a recent communication in a dental journal on the dental education of the public is worthy of emphasis. About the only dental education many communities receive is through newspaper advertisements or handbills like the following, which have appeared in print:

1. "New discovery for painless extraction of teeth by application to gums; no drugs."

2. "Dr.—— makes those improved and perfect-fitting teeth for $3, $5, $8. Extracts teeth without pain. Extracting, 25 cents; with fresh gas, 50 cents."

3. "Dr. ——, the original painless extractor of teeth, has removed to his Dental Parlors, ————. Sole proprietor of Tontine, the painless fluid. All others are imitation."

4. "Treat us fairly! If you doubt our statements, investigate. Every member of our association is an expert. He has every modern appliance to work with. He has the best materials and the best tools that money will buy. He does the best work in the world. Extracting, 25 cents; with gas or zono, 50 cents; cleaning, 75 cents; silver fillings, 75 cents; gold, according to size; very best teeth, set $8."

5. "Free to all—all operations known to modern dentistry performed in most workmanlike manner—gold foil and artificial teeth at actual cost—amalgam or silver, white, tin, and gutta-percha fillings free—that is the first time such an opportunity has ever been presented to every one to save those important organs (the teeth) free of cost—old and experienced demonstrators in charge—the same decorum will be obtained as in private office, etc."

We condense the following thoughts in the communication referred to, which fully meet our approval.

"Think of the thousands of valuable teeth that will be lost simply because the people are not aware of the fact that ninety-nine per cent. of all the teeth can practically be saved. . . . Many worthy people are led to believe that painless extracting is the height of the dentist's ambition and as they are ignorant regarding the many practical methods of saving their teeth, have them all extracted. One such 'painless dentist' can destroy more teeth than a dozen conscientious dentists can save, and still we sit by and allow it to go on without a scratch of a pen to tell the people their best course. . . . At present if an individual dentist should take steps toward publishing, in any way, information concerning the care and restoration of the teeth he would be looked upon as taking an undue advantage of his fellow practitioners, and perhaps be brought before his society as a violator of the code of ethics. . . . Keep valuable information constantly before the public. . . . If the people of the community after having such a chance to inform themselves would not do so, and profit by the advice, we, the much-abused conscientious class of dentists, could then extract molars on request without feeling that we had robbed the patient of fifty dollars' worth of tooth-structure without an effort to save the same. By this method, previously stated, we can inform the people that our true calling in life is to save and restore the natural teeth, and not to destroy them by extracting as so many people suppose, and that one of our main objects is to prevent and relieve pain and suffering and not to inflict it." R. G.

